# Cognitive Performance After Kidney and Liver Transplantation: A Cross-Sectional Study Using Addenbrooke’s Cognitive Examination III

**DOI:** 10.3390/jcm15114251

**Published:** 2026-05-31

**Authors:** Maciej Miarka, Aleksandra Golenia, Piotr Olejnik, Magdalena Grusiecka-Stańczyk, Jolanta Małyszko, Joanna Raszeja-Wyszomirska, Marta Zalewska

**Affiliations:** 1 Department of Hepatology, Transplantology and Internal Medicine, Medical University of Warsaw, 02-097 Warsaw, Poland; maciej.miarka@wum.edu.pl (M.M.); magdagrusiecka@gmail.com (M.G.-S.); 2 Department of Neurology, Medical University of Warsaw, 02-097 Warsaw, Poland; aleksandra.golenia@wum.edu.pl; 3 Doctoral School, Medical University of Warsaw, 02-091 Warsaw, Poland; piotr.olejnik@wum.edu.pl; 4 Department of Nephrology, Medical University of Warsaw, 02-097 Warsaw, Poland; jolanta.malyszko@wum.edu.pl; 5 Department of Environmental Hazard Prevention and Allergology, Medical University of Warsaw, 02-097 Warsaw, Poland; marta.zalewska@wum.edu.pl

**Keywords:** liver transplantation, kidney transplantation, alcohol-associated liver disease, cognitive impairment

## Abstract

**Background**: Cognitive impairment is increasingly recognized as a clinically relevant outcome in transplant medicine. We compared multidomain cognitive performance between kidney transplant (KTx) and liver transplant (LTx) recipients and explored etiology-specific differences within the LTx cohort using a standardized cognitive assessment. **Methods**: This single-center cross-sectional study included 180 adult transplant recipients, 90 KTx and 90 LTx recipients, stratified into alcohol-associated liver disease (ALD; *n* = 25) and non-ALD etiologies (*n* = 65). Cognitive performance was assessed using Addenbrooke’s Cognitive Examination III (ACE-III). Median time since transplantation was 3.3 months in KTx recipients, 69.2 months in non-ALD LTx recipients, and 17.2 months in ALD LTx recipients. Group differences were analyzed using nonparametric tests with Holm correction, and independent predictors of global cognition were evaluated using multivariable linear regression adjusted for age, education, and time since transplantation. **Results**: Global cognitive performance differed modestly across groups, with mean ACE-III scores of 90.3 ± 8.7 in KTx, 89.3 ± 6.5 in non-ALD LTx, and 86.9 ± 12.5 in ALD LTx recipients (overall *p* = 0.047), although no pairwise differences remained significant after Holm correction. However, the relatively small ALD subgroup may have limited statistical power and may have led to underestimation of clinically relevant between-group differences. ACE-III scores ≤ 89 occurred in 30.0%, 47.7%, and 48.0% of participants, respectively, whereas scores < 82 were observed in 13.3%, 12.3%, and 20.0%. Verbal fluency was the lowest-performing domain across groups. Higher education and younger age independently predicted better cognitive performance; time since transplantation was not independently associated with cognitive outcome. **Conclusions**: Cognitive performance appeared broadly comparable across KTx and LTx recipients, but this finding should be interpreted cautiously given differences in post-transplant follow-up and potential residual confounding. Clinically relevant cognitive vulnerability was common across all groups, supporting structured multidomain assessment and longitudinal studies using harmonized methods.

## 1. Introduction

Cognitive impairment (CI), ranging from mild cognitive impairment to dementia, increasingly contributes to morbidity in older adults and is now recognized as a clinically relevant, yet still insufficiently characterized, comorbidity in solid-organ transplant candidates and recipients [1,2,3]. In end-stage kidney disease (ESKD), CI is multifactorial and reflects both vascular and non-vascular mechanisms, including cerebral small-vessel injury, uremic toxin accumulation, oxidative stress, chronic inflammation, hypercoagulability, and long-term dialysis exposure [3]. Accordingly, contemporary studies indicate a substantial burden of CI both in hemodialysis patients and among kidney transplant recipients [4,5].

In end-stage liver disease (ESLD), CI is also well recognized, although it remains less comprehensively characterized [6]. Interpretation of the available evidence is limited by substantial heterogeneity in cirrhosis etiology, definitions of hepatic encephalopathy and CI, and methods of cognitive assessment. Notably, a systematic review by Siddiqui et al. identified 43 distinct neuropsychological tests across 24 studies of liver transplant recipients with prior cirrhosis [6]. As a result, the prevalence, pattern, and reversibility of CI in more homogeneous ESLD populations remain poorly defined [6].

More specific evidence has emerged for alcohol-associated liver disease (ALD), in which CI appears particularly prevalent and may be less fully reversible [6,7,8,9]. Persistent cognitive impairment after transplantation in this population may reflect not only liver-related encephalopathy but also alcohol-related structural brain injury [6,7,8,9]. Taken together, these observations suggest that post-transplant cognitive outcomes may not represent a uniform phenotype but rather reflect the combined influence of pre-transplant disease burden, patient-related vulnerability factors, and underlying disease etiology [6,7,8,9].

ACE-III provides a multidomain assessment of attention, memory, verbal fluency, language, and visuospatial function, offering a broader cognitive profile than brief global screening instruments. ACE-III total scores range from 0 to 100, with higher scores indicating better cognitive performance; in commonly used interpretative frameworks, scores above approximately 89 are generally considered within the expected range, whereas lower values suggest possible cognitive impairment depending on age, education, and clinical context. Although ACE-III is not a transplant-specific tool, it has been used in clinical populations relevant to transplant medicine, including patients with advanced liver disease and transplant candidates or recipients, and may be useful when domain-specific cognitive vulnerability is expected. Because ACE-III performance is influenced by age and education, comparison with healthy or community-dwelling populations should be interpreted in relation to normative expectations as well as disease-specific cognitive vulnerability.

Building on our previous work on cognitive impairment in kidney and liver disease [4,7,8], we aimed to characterize post-transplant cognitive outcomes using a standardized multidomain instrument. Specifically, we assessed cognitive performance using Addenbrooke’s Cognitive Examination III (ACE-III), a validated multidomain cognitive assessment tool [10], in kidney and liver transplant recipients and explored etiology-specific differences within the LTx cohort, with particular focus on alcohol-associated liver disease.

## 2. Patients and Methods

### 2.1. Study Design and Population

This single-center cross-sectional observational study was conducted within a structured cognitive assessment program at the Medical University of Warsaw. Adult kidney transplant (KTx) and liver transplant (LTx) recipients were consecutively recruited during routine outpatient follow-up visits. Only post-transplant assessments were included. LTx recipients were stratified according to the underlying liver disease etiology into alcohol-associated liver disease (ALD) and non-ALD subgroups.

Inclusion criteria were age ≥ 18 years and the ability to complete standardized cognitive testing. Exclusion criteria comprised acute neurological events, active delirium, severe psychiatric instability, or any other condition precluding reliable cognitive assessment.

### 2.2. Cognitive Assessment

Cognitive function was assessed using Addenbrooke’s Cognitive Examination III (ACE-III), a validated multidomain cognitive screening instrument evaluating five domains: attention, verbal fluency, memory, language, and visuospatial abilities [11]. The total score ranges from 0 to 100, with lower scores indicating worse performance. A validated Polish-language version of the ACE-III was used [12]. Based on previously applied thresholds in the Polish literature, scores of ≤89 were considered suggestive of mild cognitive impairment, whereas scores of <82 indicated a high probability of dementia [13]. ACE-III was selected because it enables multidomain cognitive profiling while remaining feasible for routine outpatient use. In addition to the total ACE-III score, domain-specific subscores were analyzed to characterize cognitive profiles across transplant populations. Domain-specific scores were expressed as percentages of the maximum possible score for each domain.

### 2.3. Clinical Variables

Demographic and clinical data were obtained from medical records and study documentation. Pre-transplant cognitive status and detailed pre-transplant disease severity measures were not available in a complete and standardized manner for all participants and therefore could not be included in the multivariable models. Consequently, the present study should be interpreted as an assessment of post-transplant cognitive status rather than cognitive recovery. These data included age, sex, years of education, time since transplantation, and tacrolimus whole-blood trough concentration closest to the time of cognitive assessment. Time since transplantation was calculated from the date of transplantation to the date of cognitive testing and expressed in months.

### 2.4. Ethical Approval

The study was conducted in accordance with the Declaration of Helsinki and was approved by the Bioethics Committee of the Medical University of Warsaw (approval no. KB/45/A/2025). All participants provided written informed consent prior to study participation.

### 2.5. Statistical Analysis

Continuous variables were summarized as mean ± standard deviation (SD) or median with interquartile range (IQR), as appropriate according to data distribution. Categorical variables were presented as counts and percentages. The distribution of continuous variables was assessed visually and, where appropriate, using tests of normality.

The primary outcome was the ACE-III total score analyzed as a continuous measure of global cognitive performance. Secondary outcomes included ACE-III domain-specific subscores and descriptive categories based on previously used ACE-III thresholds in the Polish literature [13].

For comparisons across the three predefined study groups, namely kidney transplant recipients (KTx), liver transplant recipients with non-ALD etiologies (LTx non-ALD), and liver transplant recipients with alcohol-associated liver disease (LTx ALD), continuous variables were analyzed using the Kruskal–Wallis test, and categorical variables using the chi-square test or Fisher’s exact test, as appropriate. When the overall group comparison was significant, post hoc pairwise comparisons were performed using the Mann–Whitney U test with Holm correction for multiple testing. Comparisons between two groups were performed using the Mann–Whitney U test for continuous variables and the chi-square test or Fisher’s exact test for categorical variables, as appropriate. Correlations between cognitive performance and continuous clinical variables were explored using Spearman’s rank correlation coefficient.

To identify factors independently associated with global cognitive performance, multivariable linear regression analysis was performed with the ACE-III total score as the dependent variable. Covariates were selected a priori based on clinical relevance and included age, education level, and time since transplantation. Additional exploratory models including treatment-related variables, such as tacrolimus whole-blood trough concentration closest to cognitive assessment, were constructed where clinically justified. Regression coefficients (β) with corresponding 95% confidence intervals (CI) and *p*-values were reported.

Analyses were performed on available cases, and no imputation was applied for missing data. All statistical tests were two-sided, and *p*-values < 0.05 were considered statistically significant. Statistical analyses were performed using SPSS Statistics, version 28.0 (IBM Corp., Armonk, NY, USA).

## 3. Results

### 3.1. Study Population

The study included 180 adult transplant recipients: 90 kidney transplant (KTx) recipients and 90 liver transplant (LTx) recipients. Among LTx recipients, 25 had alcohol-associated liver disease (ALD) and 65 had non-ALD etiologies. Baseline characteristics are summarized in Table 1. The groups differed in age and time since transplantation, whereas sex distribution and years of education were comparable. Tacrolimus whole-blood trough concentration also differed across groups.

### 3.2. Global Cognitive Performance

Global cognitive performance differed modestly across the three study groups. Mean ACE-III total scores were 90.3 ± 8.7 in KTx recipients, 89.3 ± 6.5 in LTx non-ALD recipients, and 86.9 ± 12.5 in LTx ALD recipients (Kruskal–Wallis *p* = 0.047). However, no pairwise comparisons remained significant after Holm correction.

Using previously applied ACE-III thresholds, scores suggestive of mild cognitive impairment (≤89) were observed in 27/90 (30.0%) KTx recipients, 31/65 (47.7%) LTx non-ALD recipients, and 12/25 (48.0%) LTx ALD recipients (overall *p* = 0.050). Scores indicating a high probability of dementia (<82) were present in 12/90 (13.3%), 8/65 (12.3%), and 5/25 (20.0%) participants, respectively (overall *p* = 0.625). Across the entire cohort, 70/180 participants (38.9%) had ACE-III scores ≤ 89 and 25/180 (13.9%) had scores < 82.

### 3.3. Domain-Specific Cognitive Profile

Domain-specific analysis showed a broadly similar cognitive pattern across transplant groups. Attention and language showed the highest mean scores, whereas verbal fluency was the lowest-performing domain in all groups. Mean verbal fluency scores were 82.9 ± 14.5 in KTx recipients, 80.9 ± 13.3 in LTx non-ALD recipients, and 78.7 ± 17.1 in LTx ALD recipients. Memory and visuospatial performance were intermediate, with numerically lower values in LTx ALD recipients. No between-group differences in individual ACE-III domains reached statistical significance (all *p* > 0.05). Detailed results of global and domain-specific cognitive performance are presented in Table 2.

### 3.4. Predictors of Global Cognitive Performance

In the prespecified multivariable linear regression model including age, education level, and time since transplantation, higher education was independently associated with better global cognitive performance (β = 0.95, *p* < 0.001), whereas older age was independently associated with lower ACE-III total score (β = −0.12, *p* = 0.013). Time since transplantation was not independently associated with ACE-III total score (β = −0.009, *p* = 0.552). Detailed results of the multivariable regression model are presented in Table 3.

### 3.5. Exploratory Analysis of Tacrolimus Exposure

In an exploratory analysis, tacrolimus whole-blood trough concentration was added to the multivariable model. Higher tacrolimus concentration was associated with better ACE-III total score in the overall cohort (β = 0.36, *p* = 0.010). In subgroup analyses, this association was observed in KTx recipients (β = 0.65, *p* < 0.001), but not in LTx recipients (β = −0.03, *p* = 0.94). Given the cross-sectional and exploratory nature of this analysis, this association should be interpreted cautiously and should not be considered evidence of a beneficial effect of tacrolimus on cognitive performance.

## 4. Discussion

In this cross-sectional study of kidney and liver transplant recipients evaluated at different post-transplant time points, global cognitive performance was broadly comparable across groups. Although the overall difference in ACE-III total score reached nominal statistical significance, no pairwise differences remained significant after correction for multiple testing. At the same time, the proportion of recipients with ACE-III scores suggestive of mild cognitive impairment remained clinically relevant across all study groups, indicating that cognitive vulnerability may persist despite generally favorable group-level mean scores [4,6,7,8,9].

Importantly, the absence of statistically significant pairwise differences after correction for multiple testing should not be interpreted as evidence of complete cognitive equivalence between groups, particularly given the relatively small ALD subgroup and the possibility of limited statistical power.

These findings are consistent with growing evidence that post-transplant cognitive outcomes cannot be reduced to a simple dichotomy of recovery versus non-recovery. In kidney transplantation, cognitive dysfunction has been linked to the combined effects of vascular injury, uremic toxicity, systemic inflammation, and dialysis-related factors, and may persist despite transplantation in a proportion of recipients [3,4,6]. In liver transplantation, persistent cognitive deficits have also been described, although interpretation of the literature is complicated by substantial heterogeneity in cirrhosis etiology, definitions of impairment, and methods of cognitive assessment [14,15,16]. Our findings extend this literature by showing that, when assessed with the same multidomain instrument, kidney and liver transplant recipients may exhibit broadly similar cognitive profiles after transplantation.

Verbal fluency was the lowest-performing ACE-III domain across all groups. This pattern may be clinically relevant, as verbal fluency reflects executive efficiency, processing speed, and frontal–subcortical network integrity. Persistent vulnerability of these domains has been described in both advanced kidney disease and chronic liver disease, including hepatic encephalopathy-related cognitive dysfunction [4,6,15,16,17]. In the liver disease setting, executive dysfunction may persist after transplantation, particularly in patients with prior hepatic encephalopathy or structural brain abnormalities [14,15,16]. Therefore, lower verbal fluency in transplant recipients may reflect an overlap between age-related decline, frontal–subcortical vulnerability related to chronic liver or kidney disease, prior hepatic encephalopathy in selected patients, and differences in cognitive reserve.

Within the liver transplant cohort, recipients with alcohol-associated liver disease showed numerically lower ACE-III scores and greater variability than those transplanted for non-ALD indications, although these differences were not robust after correction for multiple comparisons. This observation should therefore be interpreted cautiously. Previous work from our group demonstrated that cognitive impairment is particularly frequent in ALD before transplantation and may remain substantial after liver transplantation in a subset of recipients [7,8,9]. More broadly, chronic alcohol exposure has been associated with persistent deficits across multiple cognitive domains and with structural brain injury that may limit reversibility of cognitive dysfunction [18,19,20].

Interpretation of etiology-specific findings is further limited by differences in post-transplant follow-up. In our dataset, follow-up was substantially longer in LTx recipients than in KTx recipients, and within the LTx cohort it was shorter in ALD than in non-ALD recipients. Accordingly, the present design does not allow firm conclusions regarding organ-specific recovery trajectories or the independent effect of liver disease etiology on long-term cognition. The numerical differences observed between LTx subgroups should therefore be considered hypothesis-generating rather than definitive [14,15,16,18,19,20]. A study design with harmonized post-transplant follow-up intervals would be better suited to determine whether the observed numerical differences reflect persistent etiology-related vulnerability, differential recovery, or late cognitive decline.

Higher educational attainment was independently associated with better ACE-III total score, whereas older age was associated with worse cognitive performance. The association between older age and lower ACE-III score is consistent with previous evidence showing age-related decline in several cognitive domains, particularly processing speed, executive function, memory, and verbal fluency. These findings are consistent with the concept of cognitive reserve and suggest that patient-related vulnerability factors may substantially modify post-transplant cognitive outcomes [4,6,20]. Education may be particularly relevant for verbal fluency because this domain depends not only on executive retrieval strategies but also on vocabulary, lifelong language use, and cognitive reserve. Higher educational attainment may therefore support better verbal fluency through more efficient semantic organization and more frequent engagement of language-related networks. By contrast, time since transplantation was not independently associated with cognitive performance, indicating that elapsed time alone may be an insufficient surrogate of cognitive recovery in the absence of longitudinal within-patient assessment.

An exploratory association between higher tacrolimus levels and better cognitive performance was observed, particularly in KTx recipients. This finding may appear counterintuitive, as tacrolimus is a well-recognized cause of neurotoxicity, especially at high concentrations or in the early post-transplant period. In the present cross-sectional analysis, tacrolimus concentration may have acted as a surrogate marker of other clinical factors, including closer post-transplant monitoring, better adherence, shorter time from transplantation, or selection of clinically stable patients. Therefore, this association should not be interpreted as evidence of a beneficial cognitive effect of tacrolimus, but rather as a hypothesis-generating observation requiring longitudinal validation. Evidence from non-transplant populations exposed to immunosuppressive therapy suggests that cognitive outcomes may be influenced not only by the drug itself but also by the underlying inflammatory or autoimmune disease, cumulative glucocorticoid exposure, comorbidities, and treatment intensity. Therefore, disentangling the independent cognitive effects of immunosuppression from surgery, organ failure, and the post-transplant clinical course remains challenging.

This study has several strengths, including the use of the same multidomain cognitive instrument in two solid-organ transplant populations and etiology-specific stratification within the LTx cohort, which is relevant in view of the growing burden of ALD in transplant practice. Its limitations should also be acknowledged. The cross-sectional design precludes assessment of individual cognitive trajectories; post-transplant follow-up intervals differed substantially between groups; and ACE-III is a screening rather than a full neuropsychological instrument. Furthermore, pre-transplant cognitive status and detailed pre-transplant disease severity measures were not available in a complete and standardized manner for all participants, limiting our ability to distinguish post-transplant cognitive status from true cognitive recovery.

It should also be acknowledged that kidney transplant recipients are typically exposed to kidney replacement therapy, including dialysis, before transplantation, which may have independently influenced cognitive function and further complicates direct comparison with liver transplant recipients. Finally, the single-center design and the relatively modest sample size, particularly within the ALD subgroup, may limit generalizability and increase susceptibility to residual bias.

The present findings indicate broadly comparable cognitive performance across transplant groups, despite a substantial proportion of recipients showing clinically relevant cognitive vulnerability. Rather than suggesting a uniform post-transplant cognitive phenotype, the results support the concept that cognitive outcomes reflect the combined influence of pre-transplant disease burden, patient-related vulnerability factors, and, in the case of ALD, potentially less reversible alcohol-related and hepatic brain injury [7,8,9].

## 5. Conclusions

Cognitive performance was broadly comparable across kidney and liver transplant recipients, with no clear between-group differences after correction for multiple testing. However, a substantial proportion of recipients had ACE-III scores suggestive of cognitive impairment, indicating that clinically relevant cognitive vulnerability may persist despite generally favorable group-level performance. These findings support the incorporation of structured multidomain cognitive assessment into post-transplant follow-up and highlight the need for longitudinal studies using harmonized cognitive tools and comparable post-transplant time points, particularly in alcohol-associated liver disease.

## Figures and Tables

**Table 1:** Baseline characteristics of the study population.

Characteristic	KTx (*n* = 90)	LTx Non-ALD (*n* = 65)	LTx ALD (*n* = 25)	*p*-Value
Age, years	51.0 ± 12.3	55.9 ± 14.0	55.4 ± 10.1	0.016
Female sex, *n* (%)	38 (42.2)	28 (43.1)	5 (20.0)	0.100
Education, years	14.3 ± 3.4	14.0 ± 3.0	12.9 ± 3.1	0.182
Time since transplantation, months	3.3 [1.8–17.4]	69.2 [22.7–107.7]	17.2 [4.5–59.7]	<0.001
Tacrolimus whole-blood trough concentration, ng/mL	12.6 ± 4.5	6.2 ± 3.0	6.3 ± 2.3	<0.001

Data are presented as mean ± SD, median [IQR], or *n* (%), as appropriate. Overall group comparisons were performed using the Kruskal–Wallis test for continuous variables and the chi-square test for categorical variables. KTx, kidney transplantation; LTx, liver transplantation; ALD, alcohol-associated liver disease.

**Table 2:** Global and domain-specific cognitive performance assessed with ACE-III.

Cognitive Measure	KTx (*n* = 90)	LTx Non-ALD (*n* = 65)	LTx ALD (*n* = 25)	*p*-Value
ACE-III total score (points)	90.3 ± 8.7	89.3 ± 6.5	86.9 ± 12.5	0.047
ACE-III ≤ 89, *n* (%)	27 (30.0)	31 (47.7)	12 (48.0)	0.050
ACE-III < 82, *n* (%)	12 (13.3)	8 (12.3)	5 (20.0)	0.625
Attention (%)	94.5 ± 7.3	93.7 ± 8.6	93.0 ± 9.3	0.616
Memory (%)	88.3 ± 12.9	86.2 ± 11.8	83.0 ± 17.3	0.115
Verbal fluency (%)	82.9 ± 14.5	80.9 ± 13.3	78.7 ± 17.1	0.385
Language (%)	94.1 ± 8.1	94.2 ± 6.2	93.1 ± 11.2	0.840
Visuospatial abilities (%)	89.3 ± 13.3	88.8 ± 9.6	84.3 ± 16.5	0.068

Data are presented as mean ± SD or *n* (%). Overall group comparisons were performed using the Kruskal–Wallis test for continuous variables and the chi-square test for categorical variables. ACE-III scores ≤ 89 were considered suggestive of mild cognitive impairment, and scores < 82 indicated a high probability of dementia. ACE-III, Addenbrooke’s Cognitive Examination III; KTx, kidney transplantation; LTx, liver transplantation; ALD, alcohol-associated liver disease.

**Table 3:** Multivariable linear regression analysis for ACE-III total score.

Variable	β	95% CI	*p*-Value
Age, years	−0.12	−0.22 to −0.03	0.013
Education, years	0.95	0.58 to 1.31	<0.001
Time since transplantation, months	−0.009	−0.030 to 0.030	0.552

Model characteristics: R^2^ = 0.202; adjusted R^2^ = 0.184; overall model *p* < 0.001. ACE-III, Addenbrooke’s Cognitive Examination III; CI, confidence interval. β coefficients represent unstandardized regression coefficients.

## Data Availability

The data supporting the findings of this study are available from the corresponding author upon reasonable request. The data are not publicly available due to privacy and ethical restrictions.
